# Return to work after sick leave due to musculoskeletal disorder or injury: a longitudinal study conducted in Brazil

**DOI:** 10.1186/s12889-023-16789-z

**Published:** 2023-09-28

**Authors:** João Silvestre Silva-Junior, Maria Carmen Martinez, Felipe Seiti Sekiya, Cristiano Barreto de Miranda, Frida Marina Fischer

**Affiliations:** 1 Department of Forensic Medicine, Bioethics, Occupational Medicine and Physical and Rehabilitation Medicine, University of São Paulo Medical School (FMUSP), São Paulo, Brazil; 2 Department of Medicine, São Camilo University Center, São Paulo, Brazil; 3 Occupational Medicine Residency Program, Clinics Hospital, University of São Paulo Medical School (FMUSP), São Paulo, Brazil; 4 WAF Informatics and Health, São Paulo, Brazil; 5 General Coordination of Occupational Health Surveillance, Department of Environmental Health Surveillance and Occupational Health, Secretariat for Health and Environment Surveillance, Ministry of Health, Brasília, Brazil; 6 Department of Environmental Health, School of Public Health, University of São Paulo, São Paulo, Brazil

**Keywords:** Return to work, Musculoskeletal system, Longitudinal study, Occupational health

## Abstract

**Background:**

Musculoskeletal disorders and injuries (MSDI) are conditions that affect the locomotor system characterized by pain and impairment of functionality. They are the leading cause of years lived with disability. The aim of this study was to analyze the factors that influence the return to work (RTW) among workers on sick leave due to MSDI.

**Methods:**

A longitudinal study was conducted in the city of São Paulo, Brazil, between 2020–2022. The participants were 216 workers who required social security compensation due to MSDI. They filled out online questionnaires about their sociodemographic characteristics, health risk behaviors, work characteristics and health conditions. They were followed for 365 days after their first day of sick leave. A Cox regression was performed to identify the factors that influenced their first RTW.

**Results:**

Most participants were male (53.0%), mean age was 39.5 years (SD 10.6), 70.4% returned to work within the one-year follow-up period. The mean duration of sick leave was 192.6 days. Factors associated with a lower RTW were age 40 years and older (HR 0.54; 95%CI 0.39–0.76) and the interaction between perceptions of the need for improvement in the physical and psychological domains of quality of life (HR 0.67; 95%CI 0.48–0.94).

**Conclusions:**

Occupational healthcare professionals should pay greater attention to patients who are aging and those with perceived worse physical and psychological conditions, in order to facilitate the reintegration process and promote sustained RTW after sick leave due to musculoskeletal disorder or injury.

## Introduction

According to the World Health Organization (WHO), musculoskeletal disorders and injuries (MSDI) are pathological conditions that affect the structures of the locomotor system, including bones, muscles, joints, and connective tissue. These conditions are characterized by pain and reduced mobility, dexterity, and functionality, which can lead to impaired work performance. They can have either a sudden onset with limited duration or a chronic progression that lasts throughout an individual's life, potentially causing limitations and disabilities [1].

The Global Burden of Diseases study reports that approximately 1.71 billion people worldwide are affected by MSDI. These conditions are the leading cause of Years Lived with Disability (YLD), accounting for 17% of all causes. The most prevalent forms of MSDI include low back pain, bone fractures, and osteoarthritis [2].

Various factors contribute to the development of MSDI, which can be classified as individual, biomechanical, and psychosocial factors. Individual factors include a sedentary lifestyle, smoking, high body mass index (BMI) and presence of comorbidities [3]. Biomechanical risk factors involve physical exertion, repetitive movements, improper static or dynamic posture and heavy lifting [3]. Psychosocial factors, particularly those related to work, include low social support, job dissatisfaction, high occupational stress, and lack of control at work [3].

Depending on the degree of dysfunction caused by MSDI, individuals may experience partial or total reduction in work capacity, either temporarily or permanently. In Brazil, disability benefits are provided to workers who require sick leave for more than 15 days. Between 2017 and 2019, an average of 2.32 million benefits were granted, with approximately 662,000 attributed to MSDI, comprising 40% of diseases and 60% of injuries [4]. After the cessation of this benefit, workers are required to report back to their companies for a return-to-work process, with necessitates evaluation by an occupational medicine professional if the sick leave exceeds 30 days.

Recent longitudinal studies conducted in several northern-hemisphere developed countries identified factors influencing the rate of return to work (RTW) in individuals with MSDI. These factors include: sociodemographic characteristics (such as sex [5–7], age [5, 7, 8], marital status [7], and education level [5, 7, 9]), working conditions (such as physical exertion [5, 6, 10, 11], length of absence [12], social support [8, 11], and self-efficacy [8, 11, 13, 14]) and clinical characteristics (such as site of injury [5], pain intensity [5, 11], comorbidities [7, 8, 12], and treatment used [6, 11]).

Considering the epidemiological significance of MSDI, the impact of these conditions on workers’ functionality, and their individual and social costs, it is in essential to identify the factors that predict RTW among workers on sick leave due to such conditions in the Brazilian context. This information will not only help identify individuals who require more attention in the return process, but also aid in defining more effective strategies for rehabilitation and professional reintegration. The aim of this study was to analyze the sociodemographic, occupational, and clinical factors that influence the RTW after sick leave due to musculoskeletal disorders or injuries.

## Methods

### Design

This longitudinal analytical study was conducted between 2020 and 2022.

### Location

A social security service unit in the city of São Paulo was selected based on convenience. São Paulo is the most populous city in Brazil, located in the southeastern region of the country.

### Sampling

In Brazil, if a worker´s sick leave lasts up to 15 days, the employer is responsible for providing payment. However, if the sick leave exceeds 15 days, workers must claim sickness benefits from the public social security system, known as the National Social Security Institute (INSS). Applicants undergo medical evaluations to determine eligibility for sick leave benefits. For this study, participants were workers on long-term sick leave for more than fifteen days who had sought benefits due to disability for work.

The calculation of the sample size required to achieve a dependable estimate of the proportion within an unrestricted population was based on the estimated prevalence of individuals engaging in their 'first return to work after a prolonged absence due to musculoskeletal disorders,' as reported in the study conducted by Kausto et al. [10]. A confidence level of 95% (α = 0.05) was selected, corresponding to a critical value of 1.96, while maintaining a maximum acceptable estimation error of 5%. The resulting minimum sample size determined for this study is 114 participants.

### Inclusion and exclusion criteria

The inclusion criteria for the study were as follows: participants needed to have a formal employment contract with a permanent work situation, and they needed to have submitted an application for sickness benefit due to an illness classified in chapter XIII (Diseases of the musculoskeletal system and connective tissue) or chapter XIX (Injuries, poisoning and some other consequences of external causes) of the 10^th^ edition of the International Statistical Classification of Diseases and Health-Related Problems (ICD10). Requests resulting from Mononeuropathies of the Upper Limbs (ICD10 G56) were also included, as their risk factors and clinical courses are similar to musculoskeletal disorders, although they are not strictly classified as such.

Workers without formal employment or with multiple formal employment were excluded due to the potential variability in study outcome. Individuals with a history of returning to work before their participation in this current study were also excluded.

### Data collection

After undergoing a medical examination, workers who met the inclusion criteria were taken to a separate room, where they were invited to participate in the study and received information about its objectives and potential risks. Out of the 248 workers who were invited, 216 met the criteria and voluntarily agreed to participate. All of them signed a free and informed consent statement. They were then invited to complete a questionnaire using a link to an online platform.

### Instruments

The participants answered a questionnaire consisting of four blocks of questions:

I. Sociodemographic characteristics: This block included questions about sex, date of birth, schooling and marital status;
II. Health risk behaviors: Participants were assessed for smoking, using the Fagerström Tolerance Questionnaire [15]; harmful alcohol consumption was assessed using the Audit C questionnaire developed by the World Health Organization [16]; and physical activity in the weeks prior to going on sick leave was also assessed;
III. Characteristics and conditions of work: This block included questions about the participant´s current job position, number of years working in the current company, entry time for work shift, and occupational psychosocial factors evaluated using the Brazilian Portuguese version of the Effort-Reward Imbalance Questionnaire [17];
IV. Clinical condition: This block comprised questions regarding clinical morbidities under medical follow-up; as well as self-related weight and height for calculating body mass index (BMI); and the Brazilian Portuguese version for the WHOQOL-bref (World Health Organization Quality of Life) questionnaire with 26 questions and answers on a 5-points Likert Scale. From this questionnaire, the participant´s perception of quality of life and satisfaction with health were analyzed by a single question; and mean scores from questions about physical (7), psychological (6), social relationships (3), and environmental (8) domains indicated a category – “need to improve”, “regular”, “good”, or “very good” [18].

After each participant completed these questionnaires, one of the researchers accessed their expert medical report to obtain additional information, including the Brazilian Classification of Occupations (BCO) code, the ICD10 code for the reason for sick leave, the type of benefit granted (work-related/ no work-related / not reported) and the date of onset of the disability.

### Outcome

The date of first RTW was obtained from the National Register of Social Information (CNIS), a national system that collects workers’ social security data. The outcome, referred to as “survival time”, was evaluated as the number of days between the date when the work disability condition started (according to the medical expert report) and the date of the first RTW in the same company where the participant was working at the time of going on sick leave. In cases where no RTW occurred during the follow-up period, the end of the follow-up was defined as 365 days after the first day of work disability, representing type I right censoring.

### Analysis

The descriptive analyses included calculation means and standard deviations (SD) for quantitative variables, and absolute and relative frequencies for qualitative variables. For survival analyses (Kaplan–Meier curves and Cox regression), variables with categories that had a small number of participants were dichotomized.

Kaplan–Meier survival curves were constructed to estimate the probabilities of survival overall and accordance to the evaluated covariates. The log-rank test was used to compare accumulated survival curves between categories of independent variables. The statiscal significance was considered when *p* < 0.05. Those variables presenting *p* < 0.20 in the univariate analysis were selected for multiple analysis to avoid excluding any variable with potential confounding or interaction effects. 

Cox regression analysis with the stepwise forward method was used to evaluate the combined effect of independent variables on the outcome. The assumption of risk proportionality was assessed using residual graphs and the Schoenfeld test, and significance was considered when *p* < 0.05. Independent variables that did not meet this fundamental assumption were excluded. Although a convenience sample may introduce some challenges and limitations when applying statistical inference, hazard ratios (HR) are frequently utilized in survival analysis to compare hazard rates between different groups. The limitations of utilizing this statistical inference in the present study are explored towards the end of the discussion section.

HR values below 1.0 indicate a reduced likelihood of returning to work based on the length of the follow-up period. Possible interaction or confounding effects were tested whenever the inclusion of a new variable in the model resulted in changes in the parameter estimates (changes exceeding 10.0% in HR values and their confidence intervals, reversal of statistical significance, or parameter adjustments regardless of significance). In these situations, potential interaction/confounding effects were examined through stratified analysis. If the stratified analysis suggested interaction, the modification of the effect was also tested by creating an interaction variable (product-term considering the exposure variable and the potential interaction factor). Possible overfitting effects resulting from association/collinearity between variables were also tested.

### Ethical issues

The study was approved by the Ethics Committee for Research on Human Beings of the School of Public Health of the University of São Paulo (opinion report n^o^. 3,166,922/2019; CAEE 87446718.9.0000.5421).

## Results

Data from 216 individuals were collected. In terms of sociodemographic characteristics, it was observed that 53.2% were men. The age range was between 18.6 and 64.2 years, with a mean of 39.4 years (SD 10.6). The most common level of education was high school, with education ranging from more than 8 years to 11 years of study, accounting for 50.9% of the participants (Table 1).

**Table 1:** Distribution of the participants in sick leave due to musculoskeletal disorder or injury according to demographic characteristics and health risk behaviors, São Paulo—Brazil, 2020 (*N* = 216)

Variables	N	%
Sex		
Male	115	53.2
Female	101	46.8
Age group (in years)		
18.0 to 29.9	48	22.2
30.0 to 39.9	67	31.0
40.0 to 49.9	59	27.3
50.0 to 64.9	42	19.4
Marital status		
Married/cohabiting	117	54.2
Single	74	34.2
Separated/divorced	21	9.7
Widowed	4	1.9
Education		
Elementary school incomplete	36	16.7
Elementary school complete	27	12.5
High school incomplete	22	10.2
High school complete	88	40.7
Undergraduate incomplete	17	7.9
Undergraduate complete	24	11.1
Postgraduate complete	2	0.9
Smoking habits		
No	188	87.0
Very low	14	6.5
Low	6	2.8
Moderate	4	1.9
High	2	0.9
Very high	2	0.9
Alcohol consumption		
No	113	52.3
Light	62	28.7
Heavy	41	19.0
Physical activity		
3 times or more in a week	35	16.2
Less than 3 times in a week	64	29.6
No	117	54.2

Regarding reported health risk behaviors: 13.0% of the participants were smokers, 47.7% consumed alcoholic beverages, and 54.2% were sedentary in the weeks prior to going on sick leave (Table 1).

Table 2 presents information regarding the participants’ work. In terms of their occupation at the time of going on sick leave, a total of 108 BCO codes were mentioned. The most common groups were manual activities (61.6%), cleaning work (5%) and work as urban bus drivers (4.6%). The majority of participants had been working in their current role for less than five years (59.7%), with 17.6% having worked for less than a year. For 72.7% of participants, the start time of their work was always in the morning hours. The mean score for work effort was 14.0 points (SD 5.6) and the mean score for reward was 20.6 points (SD 9.4). A situation of imbalance was reported by 76.4% of the participants.

**Table 2:** Distribution of the participants in sick leave due to musculoskeletal disorder or injury according to occupational information, São Paulo—Brazil, 2020 (*N* = 216)

Variable	N	%
Occupation		
Administrative	15	6.9
Customer service	61	28.2
Manual	133	61.6
Supervision	7	3.2
Time in the job (in years)		
Less than 1.0	38	17.6
1.0 to 4.9	91	42.1
5.0 to 9.9	51	23.6
10.0 or more	36	16.7
Time of starting work (hours)		
Morning	157	73.0
Afternoon	25	11.6
Night	12	5.6
Rotating shifts	21	9.8
Efforts at work		
Low	169	78.2
High	47	21.8
Rewards at work		
Low	188	87.0
High	28	13.0
Effort-reward imbalance		
No	51	23.6
Yes	165	76.4
Type of benefit granted		
Not work related	159	73.6
Work related	53	24.5
Not reported	4	1.9

The mean BMI value was 27.9 kg/m^2^ (SD 4.9), ranging from 18.3 to 46.3 kg/m^2^. In terms of comorbidities, 26.4% of participants reported undergoing medical follow-up for another disease in addition to the condition that led to sick leave (Table 3).

**Table 3:** Distribution of the participants in sick leave due to musculoskeletal disorders and injuries according to quality-of-life perception and clinical aspects, São Paulo—Brazil, 2020 (*N* = 216)

Variable	N	%
Perception of quality of life		
Need to improve	36	16.7
Regular	81	37.7
Good	86	40.0
Very good	12	5.6
Perception of health		
Need to improve	43	19.9
Regular	29	13.4
Good	120	55.6
Very good	24	11.1
Quality of life – physical domain		
Need to improve	148	68.5
Regular	47	21.8
Good	18	8.3
Very good	3	1.4
Quality of life—Psychological domain		
Need to improve	46	21.3
Regular	102	47.2
Good	64	29.6
Very good	4	1.9
Quality of life—Social Relationships domain		
Need to improve	42	19.4
Regular	80	37.0
Good	77	35.6
Very good	17	7.9
Quality of life—Environmental domain		
Need to improve	110	50.9
Regular	95	44.0
Good	11	5.1
Very good	0	0.0
ICD10 Chapter		
XIII—Musculoskeletal disorders	122	56.5
XIX – Injuries by external causes	94	43.5
Comorbidity		
No	159	73.6
Yes	57	26.4
Body mass index (BMI)		
Underweight	1	0.5
Healthy weight	61	28.2
Overweight	83	38.4
Obesity	67	31.0
Not informed	4	1.9

When it comes to the participant´s perception of their quality of life, the majority (54.2%) rated it as regular or in need to improve. However, 66.7% of study participants expressed a good or very good level of satisfaction with their own health. In terms of the different domains, the social relationships domain had the highest mean score (3.5; SD 0.9), while the physical domain had the lowest mean score (2.6; SD 0.9).

Regarding the causes of the participants’ sick leave, 56.5% were classified under Group M (disorders) and 43.5% under Group S-T (injuries). The most frequent causes included back pain (11.5%), intervertebral disc disorders (10.1%), wrist and hand fractures (8.7%), shoulder injuries (7.8%) and leg fractures (5.9%). Regarding the type of benefit granted, 24.5% of the requests were considered work-related conditions.

**Fig. 1:**
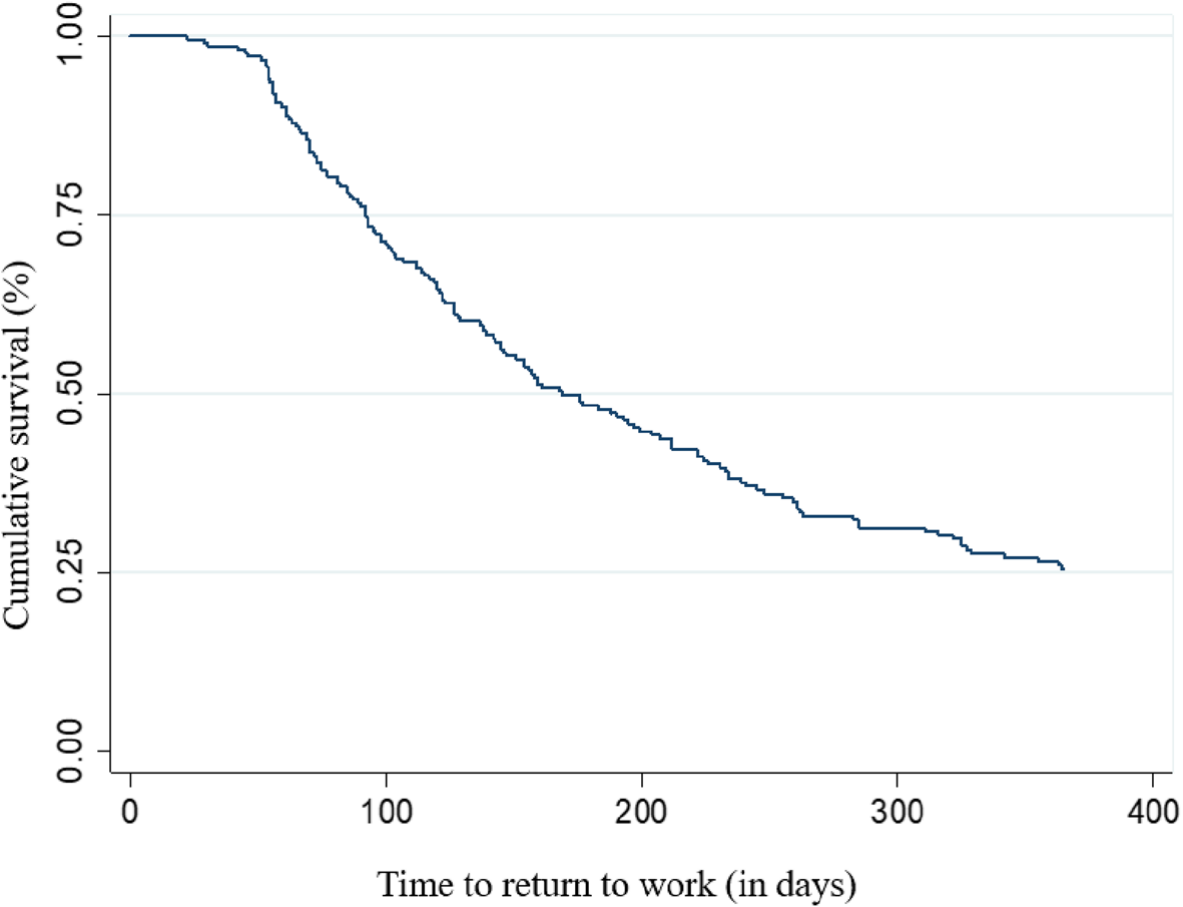
Kaplan–Meier survival curve for return to work within one year of participants on sick leave due to musculoskeletal disorder or injury. São Paulo – Brazil, 2020–2022 (*N* = 216)

The incidence of RTW was 70.4% and the mean duration of sick leave was 192.6 days (SD 118.2). Among the workers who returned to work (70.4%), the probability of remaining on sick leave for up to 90 days was 76.2%, for up to 180 days was 48.3%, for up to 270 days was 32.9%, and for up to 365 day was 25.6% (Fig. 1).

Table 4 presents the results from the univariate Cox regression analysis. It shows that several factors were significantly associated with a lower probability of returning to work, including being in the age group of 40 years or older, perceiving a regular or needing improvement in quality of life, perceiving regular or needing improvement in the physical and psychological domains of quality of life, having musculoskeletal diseases, and undergoing medical follow-up for diseases other than the condition that led to sick leave.

**Table 4:** Univariate and multiple Cox regression of return to work of participants on sick leave due to musculoskeletal disorder or injury, São Paulo, 2020–2022 (*N* = 216)

Variable	Univariate	Multiple
	HR	HR
Age group (in Years)		
Less than 40	1.00	1.00
40 or more	0.50	0.54
Perception of quality of life		
Good/very good	1.00	
Need to improve/regular	0.52	
Quality of life – Physical domain		
Good/very good	1.00	
Need to improve/regular	0.41	
Quality of life – Psychological domain		
Good/very good	1.00	
Need to improve/regular	0.64	
Interaction between physical and psychological quality of life domains		
Other conditions		1.00
Both need to improve/regular		0.67
ICD10 Chapter		
XIII—Musculoskeletal disorders	1.00	
XIX – Injuries by external causes	1.60	
Comorbidity		
No	1.00	
Yes	0.55	

*HR *Hazard ratio

The multiple analysis revealed that the age group remained significant in the model, indicating that individuals aged 40 years or older had a lower likelihood of returning to work compared to younger (HR 0.54; 95% CI 0.39–0.76). Regarding quality of life, an interaction variable between the physical and psychological domains was constructed. It showed that among individuals who perceived their condition as regular or needing improvement, there was a lower probability of returning to work within one year after going on sick leave due to MSDI (HR 0.48; 95% CI 0.48–0.94).

## Discussion

In this study, our objective was to analyze the sociodemographic, occupational, and clinical factors that influence the RTW after sick leave due to musculoskeletal disorder or injury. We identified two factors independently associated with a lower probability of returning to work: age equal or greater than 40 years and the interaction between the perceptions of needing improvement in the physical and psychological domains.

The incidence of RTW reported in previous studies with similar follow-up period has varied from 66.7% to 92% [5, 7, 8, 10]. The highest rates were observed in a survey conducted in Finland, among individuals who had been on sick leave due to back problems for at least ten days [10], and in a study conducted in Norway, among individuals who had been on sick leave for more than fifteen days due to musculoskeletal complaints [7]. The lowest rate was reported in a survey conducted in Sweden among women with an average age over 50 years [8].

The mean duration of sick leave in our study was longer than that reported in the study by Gjesdal et al. [7], where the durations were 101 days for back conditions, 110 days for upper-limb diseases, and 164 days for osteoarthritis. Another study conducted in Norway, by Rysstad et al., evaluated various trajectories between going on sick leave and reintegration into work. They found that one group returned after only 67.1 days, while another group with persistently higher sick leave duration returned to work after 221.1 days [19].

In a Norwegian cohort study with a one-year follow-up, the trajectory of individuals on sick leave due to MSDI was evaluated. It was observed that new episodes of sick leave occurred in the months following their RTW [19]. A study in Finland among workers on sick leave due to spinal problems identified recurrence factors such as advanced age, persistent health problems and comorbidities [10]. Therefore, reintegration into work is just one step in the process of returning to work, as sustained effectiveness in seeking reintegration is necessary.

### Sociodemographic characteristics

In this Brazilian study, the survival rate for the return of workers over 40 years of age was found to be lower compared to younger individuals, which is consistent with the findings of Canceliere et al. [9] and other subsequent studies [5, 7, 8]. It is likely that resolving musculoskeletal conditions in older individuals is more complex due to the structural and functional degeneration of their tissue and bone elements, which reduces their ability to respond effectively to injuries [20].

Regarding the influence of sex on the RTW, studies have yielded divergent results. Some have reported longer time off work for both men [7] and women [5, 6]. Marital status at the time of the study is also a characteristic for which there is no consensus in the literature regarding its impact on the RTW. The study by Gjesdal et al. [7] found that not having a partner favored the return, whereas in the study by Rashid, Kristofferzon and Nilsson [8] reported the opposite. In the present study, sex and marital status did not have a significant influence on the RTW. Similarly, high levels of education were not found to be significant in the present study, although previous research has suggested that high education levels are associated with a higher likelihood of returning to work [5, 7–9].

### Occupational factors

Previous studies have consistently shown that the prognosis for returning to work after a disability episode due to musculoskeletal disorders is worse for workers in manual activities [5, 6, 9–11]. This can be attributed to the higher physical and biomechanical demands faced by manual workers (blue-collar workers) compared to those in administrative occupations (white-collar workers), as well as lower levels of education and remuneration [21]. These factors may result in greater difficulties in accessing care and rehabilitation services, leading to impaired clinical resolution, and impacting work capacity recovery, especially for workers with fewer years of schooling and higher physical demands at work. However, in the present study, no significant impact was observed from the generic occupational characteristics when comparing manual workers with other occupations that likely had lower musculoskeletal workloads such as supervisors, administrative workers, and customer service workers.

Regarding organizational work characteristics (e.g., work shift) and occupational psychosocial factors (e.g., high effort, low reward, and their imbalance), the present study did not find any significant impact on the RTW outcome. This result is consistent with the findings of a review study that also did not find an association between these factors on the outcome of returning to work when studying a population with similar clinical conditions [9].

A study conducted by Devin et al. showed that individuals who received financial compensation for their time off work after spinal surgery took longer to RTW [5]. In Brazil, workers who receive a work-related type of disability benefit are entitled to job security for a minimum period of twelve months after their social security benefit ends. There was an expectation that this variable would have an impact on the rate of RTW. However, in the present study, no significant association was observed between receiving a work-related disability benefit ant the likelihood of returning to work.

### Clinical issues

In the present study, participants’ perceptions of their quality of life during sick leave were found to influence their rate of RTW. Specifically, the perception of the need for improvement in both the physical and psychological domains indicated that better management of workers’ health conditions is necessary for successful reintegration into work. This suggests a direct relationship between the disabling condition and the worker´s ability to RTW. Interestingly, occurrences of medical follow-up for other diseases, which were significant in the univariate analysis, did not emerge as a risk factor in the final model.

Previous studies have shown that factors such as diffuse muscle pain [7, 11], pain intensity levels [8], and the presence or persistence of pain after surgical treatment [22] can influence the rate of RTW. Psychological aspects, including distress, somatization, and catastrophizing, have also been identified as negative factors for RTW in the Netherlands, with a group of workers with low back pain [11]. However, in this Brazilian study, the most unfavorable situation for RTW was when dissatisfaction was present in both the physical and psychological domains, as these factors alone did not independently influence the outcome. These findings align more closely with a study conducted in Sweden, where the coexistence of depression and musculoskeletal conditions had a negative impact on reintegration to work [8].

Self-efficacy has been identified as a predictive variable for RTW in several studies [8, 11, 13, 14, 19]. This could be explained by the fact that workers’ expectation of returning to work reflect not only their confidence recovering physical abilities, but also their emotional readiness to return to the work environment and activities. Therefore, even though workers expressed need for improvement in the physical and psychological domains, they might still lack the necessary self-confidence to attempt a RTW, as observed in the current study.

It is important to note that the definition of work ability is strongly influenced by workers’ perceptions of their own condition [23]. Therefore, physical rehabilitation alone may not be sufficient. The presence of depression, which often includes feelings of worthlessness and hopelessness [24], has also identified as a negative predictor for returning to work [8, 12]. Multidisciplinary rehabilitation programs that enhance workers’ motivation have shown positive results in terms of increasing the incidence of RTW [9, 12].

### Study limitations

One of the strengths of this study was the longitudinal design for defining the outcome. However, the absence of multiple waves of data collection limited the exploration of various trajectories from the start of sick leave until the end of the follow-up period.

The study´s internal validity may have been affected by sampling bias, as participation depended on the interest of the invited individuals in the topic. However, the number of participants exceeded the minimum recommended. To address measurement bias, the study had a dual confirmation of participant´s diagnoses through medical certification from the physician who recommended the sick leave and who conducted the social security evaluation, also responsible to recruit potential cases. There may still have been some memory bias, particularly in responses to questions about exposures that had already ceased, such as work-related factors.

An additional limitation was the requirement imposed by Brazilian legislation, which only allows individuals to RTW after the end of their social security benefit. This may have led to a discrepancy between the recovery of work ability and the actual RTW date. To mitigate this bias, the outcome was determined based on registration information provided by the employing company to the Brazilian government.

The results should be interpreted with caution, as the use of convenience sampling and the relatively small number of evaluated cases may introduce biases in the results. The characteristics of the social security services and the patients make it difficult to compose a probabilistic sample. However, these exploratory results can be considered indicative of the underlying processes of the first RTW. Further studies should evaluate the progress of workers following this endeavor, taking into account the potential for recurrent absences, which could signify a lack of success in professional reintegration.

To control biases, some precautions were taken, such as using validated instruments for use in Brazil, grouping categories in variables, modeling using the stepwise method, and testing for interactions through stratified analysis, and, if necessary, by creating an interaction variable.

In terms of external validity, the findings are limited to a sample of workers from a single urban area in Brazil, although the profile observed is aligned with studies conducted in other locations.

## Conclusions

In the present study, it was identified that being 40 years and older at the time of sick leave, as well as the workers perceiving the need for improvement in both their physical and psychological domains of quality of life, had a negative impact on the RTW. Disabilities due to musculoskeletal disorder or injury led to significant financial and societal burden. Given the complexity of the return-to-work process, involving both workers and professionals who support them in a multidisciplinary approach may lead to greater success in facilitating reintegration.

More effective strategies and programs for tertiary prevention could benefit workers on sick leave in their return-to-work process, including its sustainability. Additional support should be given to individuals who are aging and those with more severe physical and psychological conditions.

## Data Availability

The datasets generated and analysed during the current study are available in the Figshare repository: https://figshare.com/articles/dataset/Silva-Junior_et_al_2023_DataSet_xlsx/21824832/3.
